# What range of extra-cardiac conduit flow velocity is detectable intraoperatively following the completion of a total cavo-pulmonary connection?

**DOI:** 10.1186/s40981-016-0054-5

**Published:** 2016-10-04

**Authors:** Satoshi Kurokawa, Kenji Doi, Shihoko Iwata, Keita Sato, Yusuke Seino, Minoru Nomura, Makoto Ozaki

**Affiliations:** Faculty of Medicine, Department of Anesthesiology, Tokyo Women’s Medical University, 8-1 Kawada-cho, Shinjuku-ku, Tokyo, ZIP 162-8666 Japan

**Keywords:** Total cavo-pulmonary connection (TCPC), Extra-cardiac conduit (ECC), ECC flow velocity, Transesophageal echocardiography (TEE)

## Abstract

**Background:**

Very few studies have investigated the blood flow velocity from the inferior vena cava (IVC) to the pulmonary artery following the Fontan operation using an extra-cardiac conduit (ECC). No studies at all have investigated the velocity immediately after the circulation is established. The purpose of this retrospective study was to find an acceptable flow velocity at the ECC following the completion of a total cavo-pulmonary connection (TCPC) via transesophageal echocardiography.

**Findings:**

We measured the mean velocity (m-V) of the blood flow proximal to the anastomosis between the IVC and ECC in eight patients and compared the results with theoretically predicted values based on assumptions regarding the cardiac output, the ratio of the IVC flow to the superior vena cava flow, and the cross-sectional form of the ECC. Mean velocities ranging from about 15 to 60 cm/s were detected in the absence of any observable stenosis. The measured m-V was significantly faster than the predicted value in our study, both collectively and in every patient individually. The shrinking and compression of the ECC might account for the faster velocities measured in our cases.

**Conclusion:**

The observed range of m-V at the ECC, about 15-60cm/s, may be acceptable for the establishment of TCPC circulation.

## Background

﻿According to a previous study, the maximum velocity of the baffle flow profile at 4.1 years on average after the completion of a total cavo-pulmonary connection (TCPC) ranged from 35 to 45 cm/s [1]. Yet, intraoperative transesophageal echocardiography (TEE) has often detected lower velocities in the extra-cardiac conduit (ECC) in subjects with well-established circulation following TCPC completion. No study has reported how fast flow in the ECC can be detected immediately after TCPC completion. The aims of this study were to compare the ECC flow velocity with the theoretically predicted values and to find an acceptable range of ECC flow velocity for the establishment of TCPC circulation.

## Methods

Eight patients who underwent TCPC completion from October 2012 to October 2013 were enrolled. This retrospective study was approved by the institutional ethics committee with a waiver of the requirement to obtain written informed consent from the parents or the guardians of the patients. Medical charts, anesthetic records, surgical records, and TEE records were retrospectively reviewed. Demographic data and ECC flow profiles observed via intraoperative TEE were collected for every patient.

The predicted mean velocity (m-V) was calculated based on the following assumptions. First, the cardiac output (CO) and body surface area (BSA) after TCPC were assumed to be correlated in the manner previously documented in healthy children [2]. Salim et al. demonstrated a good correlation between the CO and BSA in a population of 145 infants and children ranging in age from 1 day to 6.6 years [2]. Based on the regression equation obtained in their study, we calculated the CO by the following formula:

$$ \mathrm{C}\mathrm{O}=6364.022\times \mathrm{B}\mathrm{S}{\mathrm{A}}^{1/2}-1827.546 $$

Second, the inferior vena cava (IVC) flow was assumed to account for 51 % of the CO at birth, to proportionally fall to a nadir of 45 % by 2.5 years of age, and to proportionally rise to 65 %, the adult value, by 6.6 years [2]. Third, the cross-sectional area of the ECC was assumed to take the form of a perfect circle, and the ECC flow was assumed to have a flat flow profile. Based on these assumptions, the predicted m-V was calculated as follows:

$$ \mathrm{the}\;\mathrm{predicted}\ \mathrm{mV}=\frac{\mathrm{the}\ \mathrm{estimated}\;\mathrm{C}\mathrm{O}\times \mathrm{I}\mathrm{V}\mathrm{C}\;\mathrm{flow}\ \mathrm{ratio}\;\left(\%\right)\times 1/100}{1/4\times \pi {\left(\mathrm{E}\mathrm{C}\mathrm{C}\;\mathrm{diameter}\right)}^2\times 60\left( \sec \right)} $$

The ECC flow profile was obtained at the site just proximal to the IVC-ECC anastomosis. We set the sample volume at a site near the center between the ECC walls, where the color signal was most intense. The m-Vs were measured during inspiration and expiration and averaged based on an inspiration-to-expiration ratio of 1:2.

The difference between the measured m-V and predicted m-V was analyzed using a paired *t* test. Correlations of the measured m-V with the predicted m-V, BSA, and age were analyzed by Spearman’s rank correlation test. A *p* value of less than 0.05 was considered statistically significant.

## Results

**Fig. 1:**
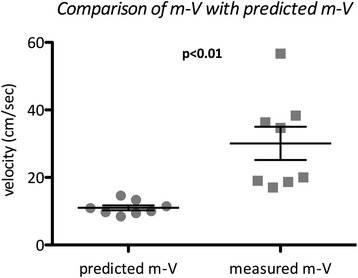
Relationship between the measured mean velocities and the predicted values. The measured m-V was significantly faster than the predicted value in our study, both collectively (*p* < 0.01) and in every patient individually

﻿﻿The BSA and age ranges of our patients were 0.46–0.72 m^2^ and 2.3–5.0 years, respectively. A staged-operation strategy was applied to all of the Fontan candidates in our institute. All 8 patients, therefore, underwent bidirectional Glenn anastomosis at 13–55 months before TCPC completion. The TCPC completion was conducted under a conventional cardio-pulmonary bypass (CPB). A 16- or 18-mm graft was applied as the ECC in all 8 patients. Hemodynamic parameters indicated well-maintained, well-established TCPC circulation in all of the patients when the TEE was measured after weaning from CPB (Table 1). The CVPs in two different territories, the superior vena cava via the internal jugular vein and the IVC via the femoral vein, were completely concordant at the time. The pre-discharge transthoracic echocardiography revealed no mosaic appearance or obvious acceleration in the flow from IVC to the PA in any of the cases. Hence, no patients showed any signs of stenosis in the IVC to the PA pathway during or after the operation. The measured m-V was significantly higher than the predicted m-V (*p* < 0.01) (Fig. 1). The measured m-V was not correlated with the predicted m-V (*p* = 0.83), patient age (*p* = 0.82), or BSA (*p* = 0.74).

**Table 1:** Demographic backgrounds of the patients studied

Case	Age (mo)	BW (kg)	BSA (m^2^)	Diagnosis	Interval from BDG (mo)	Fenestration	ECC (mm)	Predicted m-V	IVC (mm)	Pharmacol. support (γ)				sBP	CVP	rSO_2_	ScvO_2_	Measured m-V
										A	B	M	N					
1	55	13.0	0.58	SLV	47	+	18	10.9	14.3	4	3	0.5	2	85	16.5	–	74.1	20.0
2	31	12.8	0.54	SLV	14	–	18	8.5	10.0	5	2	0.5	3	95	16	–	71.4	17.0
3	28	10.4	0.48	HLHS	14	–	16	9.7	9.8	4	5	0.5	2	105	16	–	86.9	38.3
4	54	14.4	0.63	d-TGA, PS	14	–	16	14.6	9.6	5	0	0.5	3	75	13.5	81/83	81.6	34.7
5	60	18.6	0.72	TA	55	+	18	13.4	10.0	5	2	0.5	3	90	16	85/89	–	36.3
6	37	12.2	0.55	unbalanced AVSD	15	–	16	11.5	7.2	3	0	0.5	1	90	14.5	78/80	–	18.7
7	29	11.2	0.50	SLV, DORV, AVSD	13	–	16	10.0	7.0	5	0	0.5	1	80	15	86/83	–	19.0
8	27	10.0	0.46	Ebstein’s anomaly	18	+	16	9.4	5.9	4	0	0.5	1	80	15	74/67	–	56.7

*mo* months, *BW* body weight, *BSA* body surface area, *BDG* bidirectional Glenn, *ECC* extra-cardiac conduit, *m-V* mean velocity, *IVC* inferior vena cava, *pharmacol*. pharmacological, γ μg/kg/min, *A* dopamine, *B* dobutamine, *M* milrinone, *N* nitroglycerine, *sBP* systolic blood pressure, *CVP* central venous pressure, *rSO*_*2*_ cerebral regional oxygen saturation, *ScvO*_*2*_ central venous oxygen saturation, *SLV* single left ventricle, *HLHS* hypoplastic left heart syndrome, *TGA* transposition of the great arteries, *PS* pulmonary stenosis, *TA* tricuspid atresia, *AVSD* atrioventricular septal defect, *DORV* double outlet from right ventricle

## Discussion

To our knowledge, the present study is the first to elucidate an acceptable ECC flow velocity immediately after TCPC completion. The measured m-V was significantly faster than the predicted value in our study, both collectively and in every patient individually.

Two findings have the potential to invalidate the third assumption described above in calculating the predicted values and could account for the faster velocities measured in our cases: first, the discrepancy in size between the IVC and graft; second, the compression of the ECC by the patient’s heart. The former finding must have made the graft shrink at the site of the anastomosis, and the latter must have squeezed the cross-section of the ECC into a narrower form than the pure circle. Both findings are closely related to the size mismatch between the small physique of a child and the relatively large-bore graft selection to accommodate the child’s growth into an adult.

Mosbahi et al. studied the spatial distribution of the flow velocity from the right ventricular outflow tract (RVOT) to both of the branch pulmonary arteries (PAs) at three different flow rates of constant flow (low [75 %], normal [100 %], and high [125 %] versus normal baseline measurements taken in vivo) using a computational fluid dynamics model [3]. The constant flow in this model mimics Fontan circulation well in the absence of a pumping chamber driving the pulsatile pressure for pulmonary circulation. The distribution of the flow velocity in their study turned out to be quite homogenous in a straight trunk running from the RVOT to the main PA. Most of the points on the cross-sectional plane in the straight trunk had the same velocities, but a number of very thin circumferential points along the vessel wall had notably low velocities. This is quite consistent with the flat profile flow of the ECC we assumed. Mosbahi’s group also observed higher flow velocities around the corner and after the change in vessel diameters from the main PA to branch PAs and much higher velocities reaching about 2-fold the velocity in the main PA at the “high” flow rate. This condition could be easily reproduced in the presence of ECC compression and a hyperdynamic state concomitantly in our cases. Shrinkage was also unavoidable, because a 16- or 18-mm graft was selected for the ECCs for the 2- to 5-year-old children. The ECC flow velocity could have thus been closer to the IVC flow velocity and necessarily faster than the predicted velocities at the site of the anastomosis with the IVC. Given the IVC diameter range of 5.9 to 14.3 mm (median of 9.7 mm) in our patients, the IVC flow velocities could have been roughly estimated to be 3.2-fold the predicted velocities. The calculated IVC flow velocity was highest (68.8 cm/s) in our smallest patient, and a corresponding ECC velocity (56.7 cm/s) was actually observed. The ECC must also have been compressed, at least to some extent. With only negligible compression, the ECC flow velocity slows to a level close to the theoretical value due to the relative release of the shrinking. While this compression can be significant, it further enhances the elevated ECC flow velocity. These findings are likely to account for the detection of the fastest m-V in the smallest patient, the greater velocity in the present study versus the previous study, and the 2-3-fold higher m-V versus the predicted value in the remaining cases.

We extrapolate the CO in healthy subjects to our TCPC patients at the time of ECC flow measurement, that is, at about 1–2 h after separation from CPB. The CO in patients with Fontan circulation typically decreases to about 70 % of that in normal subjects at rest [4]. In other observations after the Fontan operation, Nakazawa and colleagues measured a CO of 2.45 ± 0.48 L/min/m^2^ in 10 patients with tricuspid atresia (TA) and a CO of 2.75 ± 0.72 L/min/m^2^ in another 10 patients with single ventricle physiology excluding TA [5]. Williams et al., meanwhile, found that dopamine infusion at a rate of 7.5 μg/kg/min just after the classical Fontan operation increased the cardiac index by 40 % (from 1.98 ± 0.86 to 2.75 ± 1.05 L/min/m^2^) in 9 patients [6]. Yet in both of the foregoing studies, the CO values were roughly estimated at only 50 % of the value in the healthy children investigated by Salim et al. (5.2 ± 1.4 L/min/m^2^). The hyperdynamic states in our patients must have counterbalanced the low CO typical of Fontan cases to an extent similar to that observed in the report by Williams et al. but not to an extent sufficient to permit the extrapolation of the CO of healthy children to our cases. By assuming a CO lower than the CO we actually applied in predicting the ECC m-V in the present study, the predicted m-V must be smaller. This may imply that an ECC flow of a low velocity, even a velocity lower than that previously reported, is very likely to be obtained when the shrinkage and compression of the ECC is negligible.

Fenestration was created in 3 patients in a side-to-side anastomosis fashion between the ECC and atrial wall at the mid-portion of the conduit. We measured the ECC flow at a site distal from the fenestration, so the fenestration had no effect whatsoever on the measured flow.

Our study is subject to several limitations. First, the study was retrospective. Ideally, the site, point in time, and respiratory setting for ECC flow measurement should be strictly determined. Second, we worked with only a small number of cases with widely variable backgrounds in age, diagnosis, and PA morphology. The preferred policy for intervention in recent years has been to complete the TCPC at an earlier age in life, ideally in patients younger than 3 years of age [7, 8]. A prospective investigation of a larger number of subjects with a peak age in this younger age range is therefore awaited.

## Conclusions

In conclusion, the range of ECC flow velocities widely varied in our series. Whereas a low velocity of around 15–20 cm/s was detected in most of our cases, a m-V ranging up to 60 cm/s was observed immediately after the completion of the TCPC even in the absence of significant obstruction of the ECC pathway. The observed range of m-V at the ECC, about 15–60 cm/s, may be acceptable for the establishment of TCPC circulation.

## Consent

The present study was approved by the ethics committee of Tokyo Women’s Medical University with a waiver of the requirement to obtain written informed consent from the parents or the guardians of the patients (Reference No. 3283).
